# Flexible endoscopy for pediatric tracheobronchial metallic stent placement, maintenance and long-term outcomes

**DOI:** 10.1371/journal.pone.0192557

**Published:** 2018-02-08

**Authors:** Wen-Jue Soong, Pei-Chen Tsao, Yu-Sheng Lee, Chia-Feng Yang

**Affiliations:** 1 Department of Pediatrics, Taipei Veterans General Hospital, Taipei, Taiwan; 2 Institute of Emergency and Critical Care Medicines, School of Medicine, National Yang-Ming University, Taipei, Taiwan; 3 Department of Pediatrics, Tri-service General Hospital, National Defense Medical Center, Taipei, Taiwan; University of Alabama at Birmingham, UNITED STATES

## Abstract

**Objectives:**

To assess the placement, surveillance management and long-term outcomes of the tracheobronchial (TB) balloon expandable metallic stent (BEMS) managed by therapeutic flexible endoscopy (TFE).

**Methods:**

This is a retrospective review and analysis of all computerized medical records and related flexible endoscopy videos of pediatric patients who received TB BEMS during 20 years period, from January 1997 to December 2016. TFE techniques with forceps debridement, balloon dilatation and laser ablation were used to implant stents, perform regular surveillance, maintain their functions, and expand the diameters of BEMS. Short-length (30cm-36cm) endoscopes of OD 3.2mm to 5.0mm coupled with the noninvasive ventilation, without ventilation bag, mask or airway tube, supported the whole procedures.

**Results:**

146 BEMS were implanted in 87 consecutive children, including 84 tracheal, 15 carinal and 47 bronchial stents. At the time of placement, the mean age was 35.6 ± 54.6 month-old (range 0.3–228) and the mean body weight was 13.9 ± 10.6 kg (range 2.2–60). Surveillance period was 9.4 ± 6.7 years (range, 0.3–18.0). Satisfactory clinical improvements were noted immediately in all but two patients. Seventy-two (82.8%) patients were still alive with stable respiratory status, except two patients necessitating TFE management every two months. Fifty-one stents, including 35 tracheal and 16 bronchial ones, were successfully retrieved mainly with rigid endoscopy. Implanted stents could be significantly (< .001) further expanded for growing TB lumens. The final stent diameters were positively correlated to the implanted duration. Altogether, 33 stents expired (15 patients), 51 were retrieved (40 patients), and 62 remained and functioning well (38 patients), with their mean duration of 7.4 ± 9.5, 34.9 ± 36.3 and 82.3 ± 62.5 months, respectively.

**Conclusion:**

In pediatric patients, TFE with short-length scopes coupled with this NIV support has provided a safe, feasible and effective modality in placing and subsequently managing TB BEMS with acceptable long-term outcomes.

## Introduction

Tracheobronchial (TB) narrowing in children, either congenital or acquired, that compromise breathing flow may produce significant respiratory distress and even fatal consequences. Traditionally, victims who failed the medical management need to receive more invasive surgical procedures such as tracheostomy, tracheoplasty, thoracotomy, bronchoplasty and/or extra-corporeal life support (ECLS). TB stents can be used to provide intraluminal structural support in order to maintain patency of the airways. Metallic stent placement has been introduced as an attractive option in children since 1990s, which can provide immediate, durable and stable TB lumen patency and improving quality of life [1–5]. Selected patients may benefit from this less-invasive therapy than the surgical corrections. Since stents are foreign bodies, they do have complications and associated technical problems, especially in pediatric patients. Most reported pediatric studies have been small in size and rarely given in detail on the metallic stent placement, maintenance, retrieval, and long-term outcomes [6–10].

As a quaternary referral center for complicated and difficult pediatric airway problems, we have received many patients coming from other tertiary centers locally or overseas. Our approach mainly uses flexible endoscopy (FE) for both diagnostic and various therapeutic FE (TFE) interventions, which are all supported with a technique of noninvasive ventilation (NIV) [11–12]. Since 1997, we have gradually developed novel TFE techniques for TB stent implantation with uncovered balloon expandable metal stent (BEMS) and subsequent management of associated problems. To our best knowledge, these BEMS related TFE techniques and long-term outcomes have never been reported before. The purpose of this study is to retrospectively review and analyze the placement, maintenance, retrieval and long-term outcomes of these TB BEMS in our pediatric patients.

## Material and methods

### Enrolled patients and stents

All pediatric patients who received TB BEMS from January 1997 to December 2016 in our hospital were enrolled. Computer medical records and BEMS related-FE videos were retrospectively reviewed and analyzed for their demographic data. These included patient number, age and body weight at stent placement, major underlying etiologies causing TB narrowing for stenting, locations of implanted and retrieved stents, surveillance management, stent diameters at placement, retrieval and the latest endoscopy before the end of this study, major causes of mortality, and long-term outcomes of implanted stents and patients. Our routine policy to maintain function of the implanted stent is mainly through both medical and TFE interventions. The first surveillance FE was performed in 3 to 5 days after implantation, and subsequent clinical and FE evaluations were individualized in 3 to 6 months to check and manage associated complications. The final stent diameter were measured from their last procedure of balloon dilatation plasty (BDP). Surveillance periods, varied with different outcomes, were started from the time of stent placement until 6 months after stent retrieval, patient expiring, or the end of this study.

Written informed consents, which listed 1) all possible complications and management of the implanted BEMS, FE and associated TFE were obtained from parents or guardians of the minors before procedures. The individual in this manuscript has given written informed consent (as outlined in PLOS consent form) to publish these case details. This study was approved by the “Institutional Review Board of Taipei Veterans General Hospital” (IRB-TPEVGH no.: 2014-11-004A and 2016-04-006A). The board is organized under, and operates according to International Conference on Harmonisation (ICH)/WHO Good Clinical Practice (GCP) and the applicable laws and regulations. All the medical record data were fully anonymized before access by the researchers, and the IRB waive the requirement for informed consent. This study has received grant from our hospital (V104C-190).

### Data analysis

Data were presented as mean values ± standard deviation, medians and ranges, and/or binomial percentages, when appropriate. Kaplan-Meier survival curves were plotted for outcome measures and log-rank tests were performed to analyze differences between specific subgroups. Data were analyzed and displayed with SPSS software version 21.0 (IBM-SPSS, Inc, Armonk, NY) and Prism 6.0 (GraphPad Software Inc, La Jolla, Calif).

## Results

### Patient, stent, FE and indications

**Fig 1:**
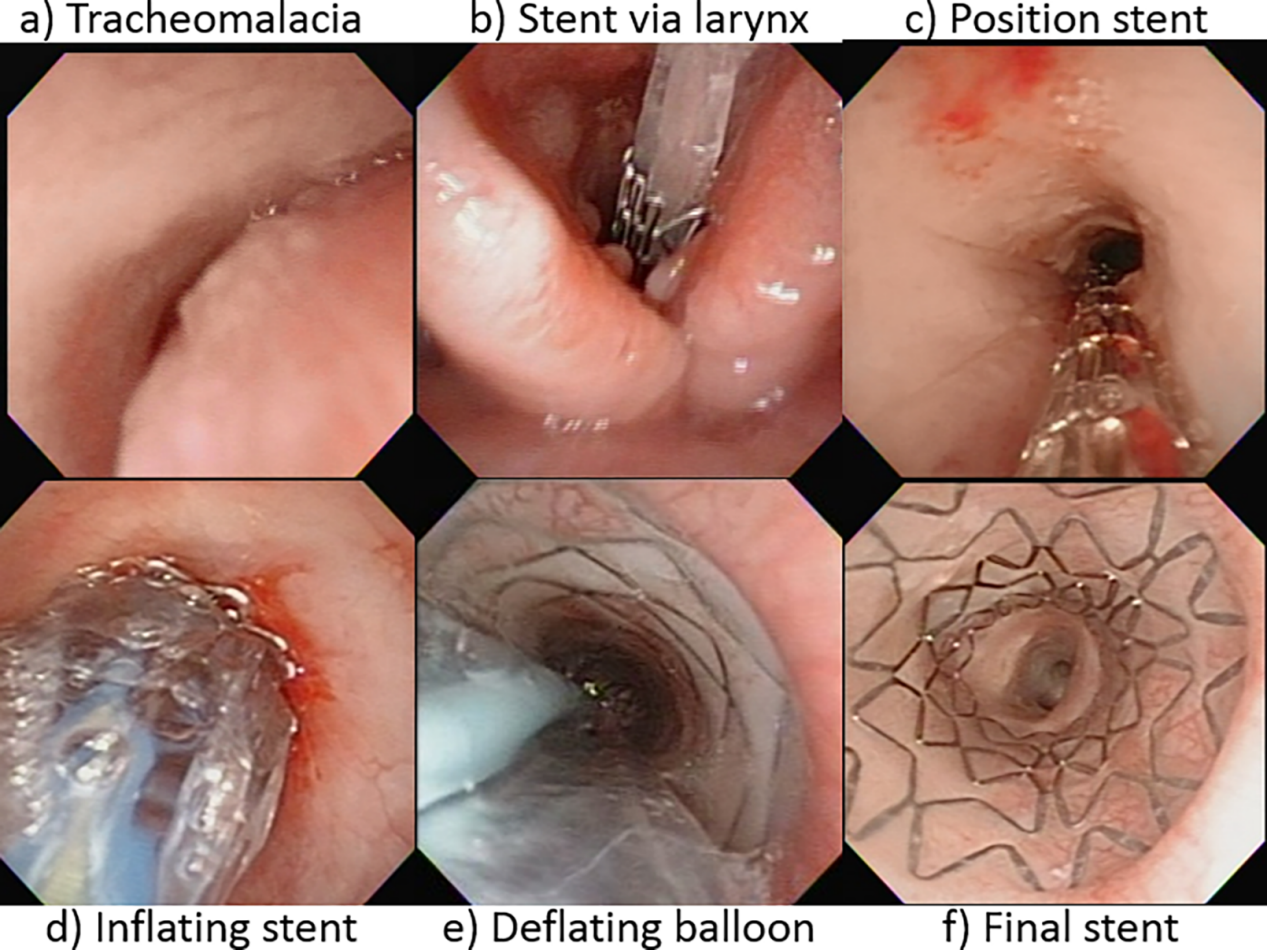
Sequential steps of stent implantation in severe tracheomalacia by flexible endoscopy. a) Tracheomalacia; b) inserting stent via larynx; c) positioning stent; d) inflating the stent; e) Deflating the balloon; f) Final stent.

During this 20-year period, a total of 146 BEMS were placed in 87 consecutive children (Table 1). Seventy-three (83.9%) patients were referred from other tertiary centers. Among them, there were 4 infants with a total of 5 stents previously placed in outside hospitals. They all were referred to us after their frequent metallic stent-related life threatening episodes due to severe obstructive tracheal granulations could not be effectively managed. We retrieved these 5 troubled-stents with rigid endoscopy (RE) and forceps, and replaced 4 new BEMS. In this study, all the BEMS placement (Fig 1 and S1 Video) and subsequent management were performed absolutely with FE, except the retrievals, in the endoscopy room which is located inside pediatric intensive care unit with full resuscitation and RE facilities. Short working-length FE of 25 cm to 36 cm with the corresponding OD 3.2 mm to 5.0 mm (Olympus; HYF-V, ENF-VQ, ENF-V2 and ENF-VT2) were used. Patients were appropriately sedated with the following NIV technique supported, without presence of any artificial ventilation bag, mask, airway tube or mechanical ventilator through the whole FE and RE procedures.

**Table 1:** Numbers of implanted tracheobronchial stents and patients.

Stent number	Patient number (%)	Subtotal (%)
1	44 (50.6)	44 (30.1)
2	33 (37.9)	66 (45.2)
3	5 (5.7)	15 (10.3)
4	4 (4.6)	16 (11.0)
5	1^a^ (1.2)	5 (3.4)
Total	87 (100)	146 (100)

^a^Patient finally expired.

### NIV technique

**Fig 2:**
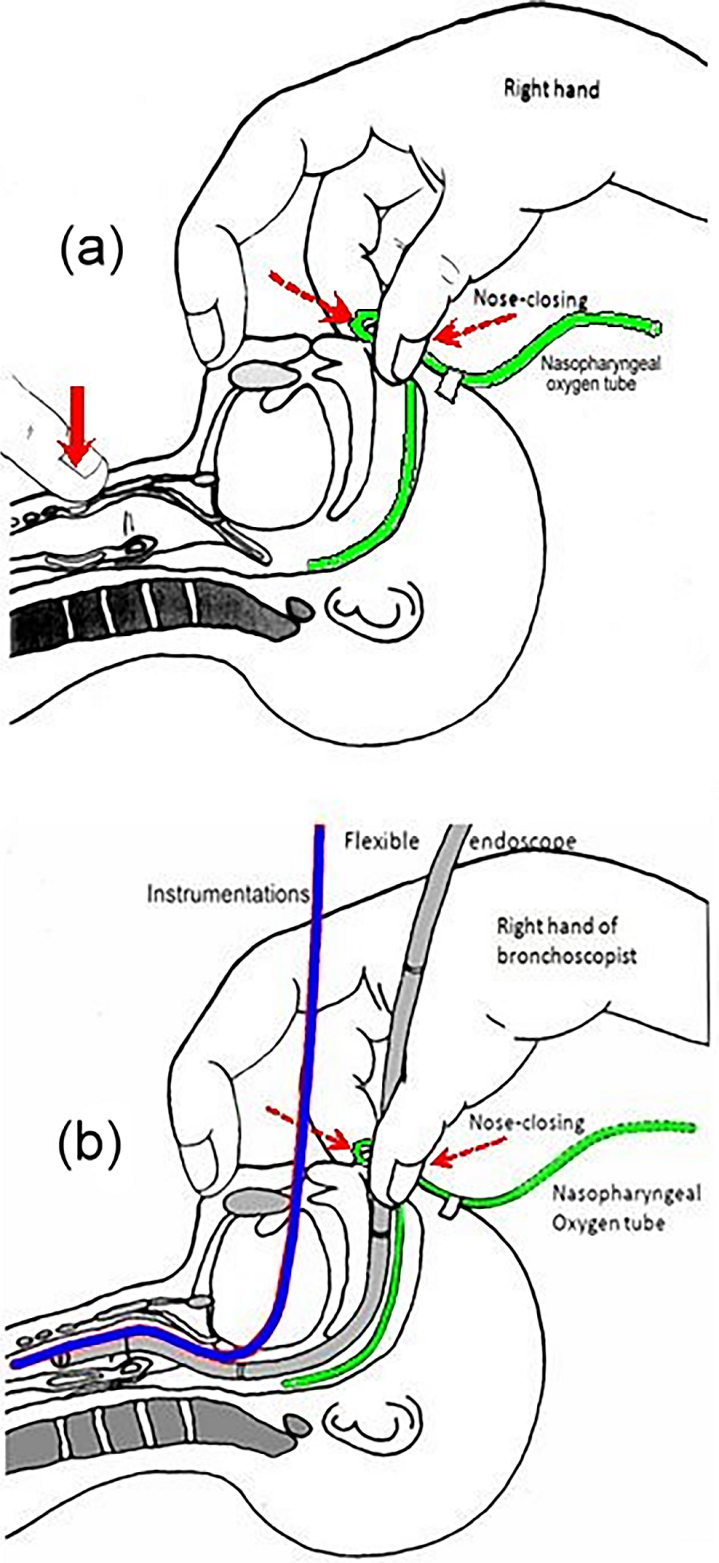
Diagram of “nasopharyngeal oxygen with intermittent nose-closure” ventilation. Done alone (a), or coupled with therapeutic flexible bronchoscopy (b).

**Fig 3:**
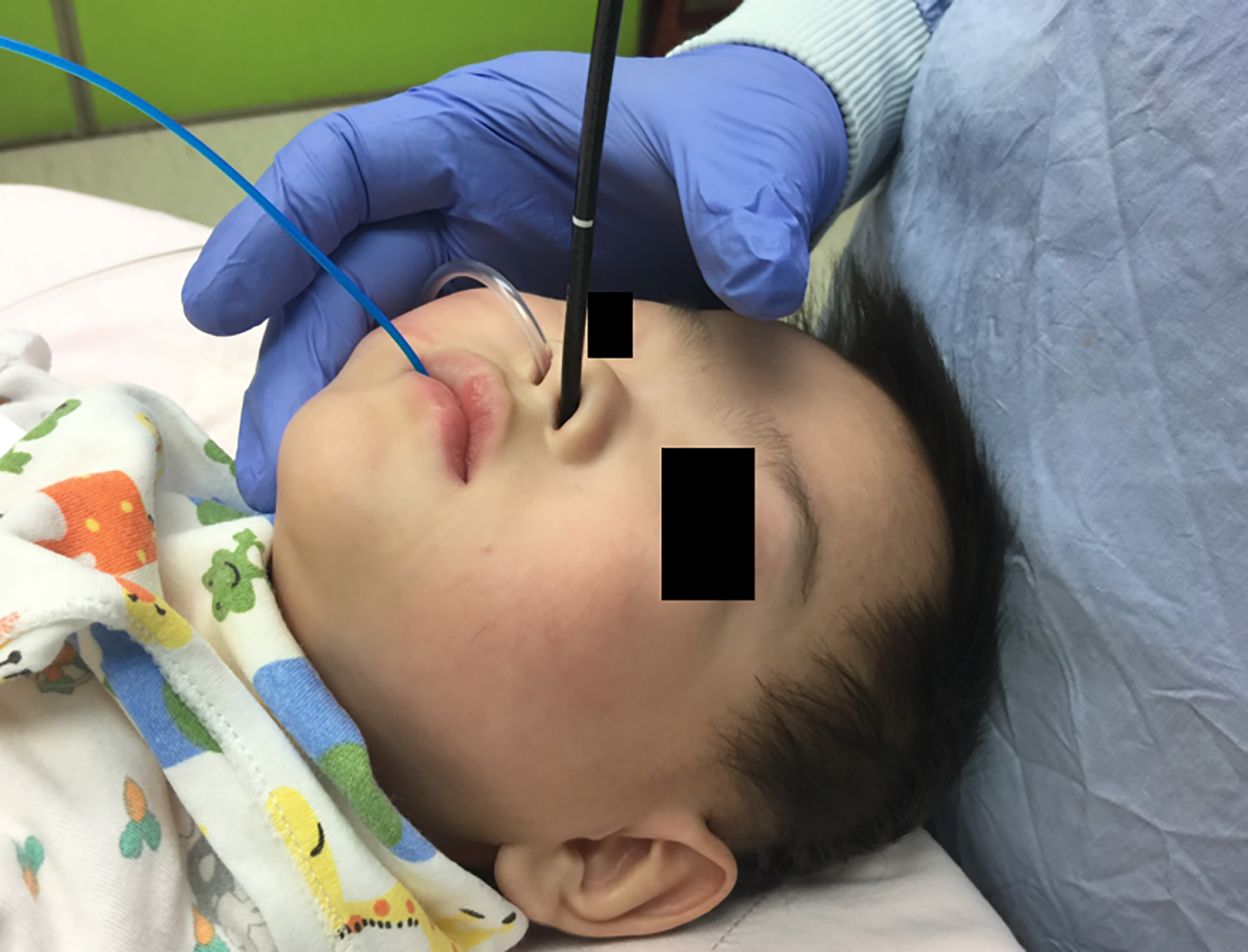
Picture of an infant undergoing therapeutic flexible bronchoscopy supported with this noninvasive ventilation maneuver. A nasopharyngeal oxygen catheter inserted into right nostril, a balloon catheter via mouth and a flexible endoscope via left nostril.

‘‘Nasopharyngeal oxygen with intermittent nose-closure and abdominal-compression, PhO_2_-NC-AC” was routinely applied for supporting oxygenation, maintaining airway patent and providing ventilation. Details of this NIV during the FE were reported before [11–12]. Briefly, it started with giving a basic continuous pharyngeal pure oxygen flow at 0.5–1.0 L/kg/minute (maximum 10.0 L/minute) via a nasopharyngeal catheter. The bronchoscopist intermittently performed nose-closure for 1.0–1.5 second as needed to create positive airway pressure and assist inspiration (Fig 2A). It was followed by passive expiration when nose closing stopped. Simultaneously, an assistant might perform abdominal-compression to generate active expiration. When the patient was in stable condition, this NIV was given optionally at a low frequency of 0–5 cycles per minute, even when the endoscope or instrument(s) still remained inside the airway (Fig 2B). During the TFE procedures, the bronchoscopist’s hand keep around the patient’s nose and mouth, that could simultaneously and easily handle the scope, instrument, and nose/mouth-close/open of this NIV (Fig 3).

When bradycardia (< 85 beats/min) or oxyhemoglobin desaturation (< 85%) developed for more than 10 seconds, more aggressive NIV was then performed. The endoscope and instrument(s) were removed from airway and pharyngeal oxygen flow was increased to 1.0 L/kg/minute (maximum 10.0 L/minute). The NIV maneuver was given at the rate of 15–20 cycles/minute. When the vital parameters returned to acceptable levels (heart rate >90 beats/min and oxyhemoglobin saturation >90%) for more than 10 seconds, the NIV returned to the previous optimal approach and the FE procedure was resumed. In case that parameters did not improve substantially after more than one minute of this aggressive NIV management, the traditional resuscitation (American Heart Association’s Pediatric Advance Life Support Guidelines) was executed. In this teamwork, the bronchoscopist was responsible for manipulating the endoscope and related instruments, and doing the nose/mouth-closure and release. This NIV support started from the preparatory period, throughout the whole FE procedure, and lasted for one minutes after finishing FE. This NIV was also applied concurrently with ECLS in 4 children requiring stent placement for their refractory severe TB malacia not ameliorated even after surgical slide tracheoplasty. Additional advantage of using this NIV was the capability of optimal expansion of both upper and low airway lumens upon the bronchoscopist’s control for better endoscopic assessment and therapeutic intervention.

To attain the success of this NIV maneuver, our flexible bronchoscopy team members were responsible for the anesthetic process through giving optimal intravenous sedation and local anesthesia, as well as monitoring closely their vital parameters throughout the whole FE /TFE procedures.

At the time of stent placement, the patient’s mean age was 35.6 ± 54.6 (range 0.3–228, median 10.5) month-old and the mean body weight was 13.9 ± 10.6 (range 2.2–60, median 10.0) kg (Table 2). The youngest age was 10 days old and the lowest weight was 2.2 kg. Surveillance information were available for all these patients with mean duration of 9.4 ± 6.7 (range, 0.3–18.0, median 8.3) years.

**Table 2:** Basic data of children with tracheobronchial stents.

Demography	Data
Stent number	146
Patient number	87
Age^a^ (month)	
Mean (SE)	35.6 (54.6)
Range, median	0.3–228, 10.5
Body weight^a^ (kg)	
Mean (SE)	13.9 (10.6)
Range, median	2.2–60, 10.0
Surveillance period (years)	
Mean (SE)	9.4 (6.7)
Range, median	0.3–18.0, 8.3

^a^At the time of stent placement

The initial 20 implanted stents in 14 patients were *Palmaz* stents (Johnson and Johnson, NJ, USA), which included one infant with 5 stents implanted. The subsequent 126 stents in 73 patients were *Intrastent* type (IntraTherapeuticsInc, MN, USA). Both types of stent have similar potential dimension ranges of 4 to 15 mm and lengths of 12 to 40 mm.

The main indication of stent placement was severe TB narrowing (malacia or stenosis) leading to prolonged endotracheal intubation or positive pressure ventilation (PPV) dependence which could not be weaned out of frequent life-threatening episodes. Other options of TFE management were either not feasible or failed. Stent placement was the last resort before undergoing more invasive surgeries. Four upper tracheal stenting were done in 4 infants to replace their tracheostomies upon parents’ request to ameliorate their frequent tracheostomy-related granulations, bleeding, and speech defect. Seven stents were placed in 4 patients who presented with severe TB collapse not corrected after previous slide tracheoplasty.

### Stent locations

**Fig 4:**
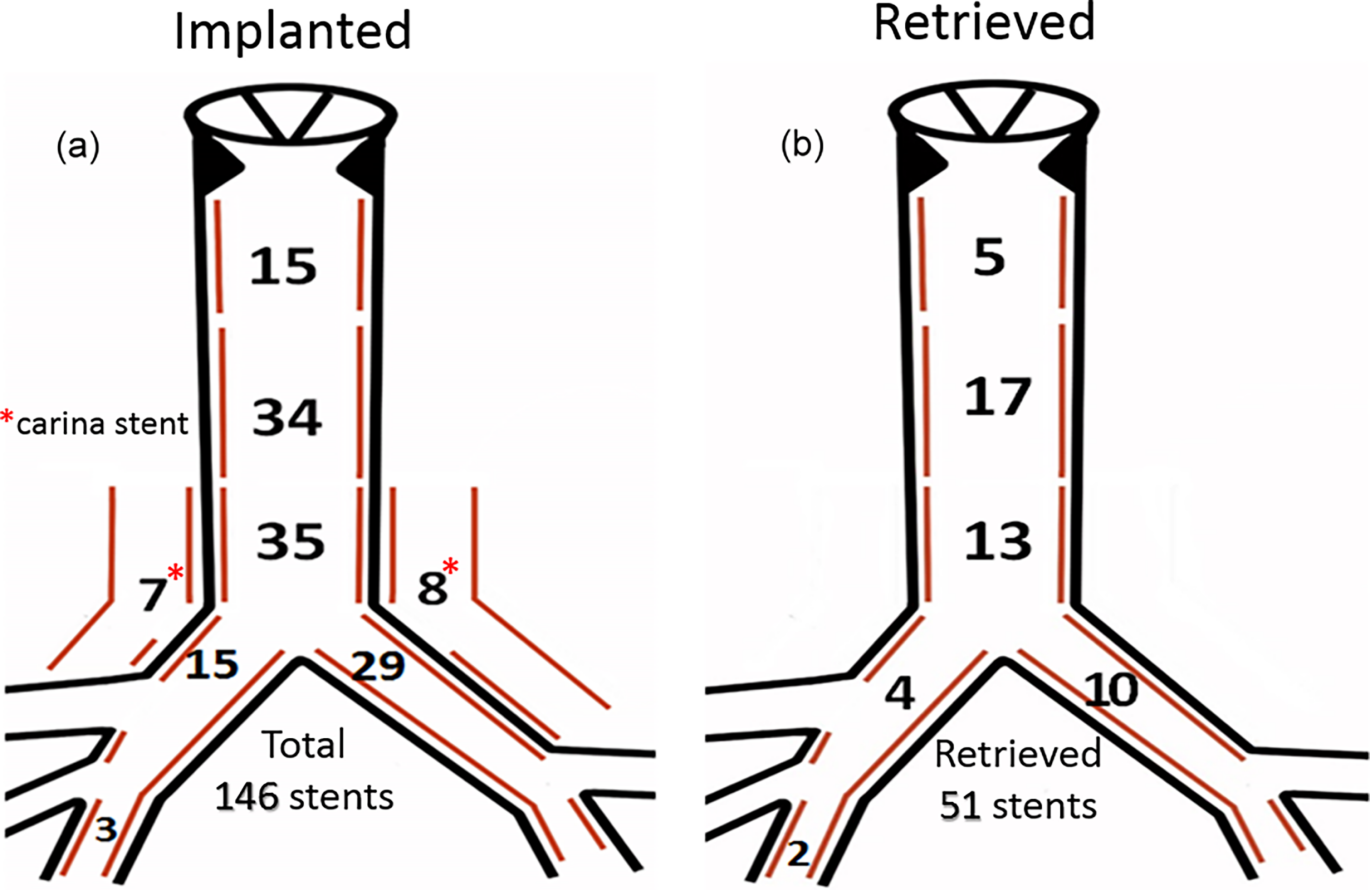
Anatomic locations of tracheobronchial stents. Implanted (a) and retrieved (b) stents.

Anatomic locations of the implanted stents are illustrated (Fig 4A). There were 84 (57.5%) stents in trachea, 15 (10.3%) stents in carina and 47 (32.2%) stents in bronchi. Carinal stents were placed specifically for correcting the peri-carinal lumen malacia. In brief, one long-length stent (8 of 30 mm and 7 of 40 mm) span from one main bronchus distally to the low trachea proximally, covering the carinal malacia region. Then, both bronchial and low tracheal portions of stent were expanded, respectively, by technique of BDP, to comply with the different diameter lumens. Finally, an artificial big side-hole (e.g. 5–6 mm in infant) was created with BDP in this carina stent toward the contralateral bronchial orifice. Similar technique had also been carried out in seven distal bronchial, 3 right and 4 left, branches. In this study, there was no any obstruction related to these carina stents. In addition, this BDP technique was applied in our center to serve the different purposes of deploying BEMS, decompressing granulations, expanding and keeping lumen patency, repairing distortion, and even making intentional destruction prior to stent retrieval.

### Major etiologies, short-term and long-term outcomes of patients

Major underlying etiologies of TB narrowing were divided into six categories (Table 3). The first leading one was the cardiovascular diseases in 39 (44.8%) patients receiving 63 (43.2%) stents. Their TB lumen narrowing was mostly secondary to the external compression which included 14 patients of severe cardiomegaly, 8 patients of pulmonary slings, 7 of complex congenital heart diseases, 6 patients of double aortic arch and 4 of aberrant subclavian artery. The second etiology was iatrogenic malacia in 13 (14.9%) patients with 19 (13.0%) stents, including 10 extremely premature infants with severe TB malacia due to prolonged endotracheal intubation and PPV [13,14]. Others causes included idiopathic, multiple anomalies, thoracic scoliosis, and tracheoesophageal fistula with esophageal atresia.

**Table 3:** Major etiologies and long-term outcomes of tracheobronchial stented patients (stent number, %).

Patient Outcomes	Cardio- vascular	Iatro- genic	Idio- pathic	Multiple anomalies	Thoracic scoliosis	TEF^a^ and EA	Total
Patient number	39 (63)	13 (19)	11 (22)	8 (19)	8 (12)	8 (11)	87 (146, 100)
Mortality	8 (18)	3 (6)	1 (3)	1 (2)		2 (4)	15 (33, 22.6)
Cause							
Respiratory		1 (3)	1 (3)	1 (2)		1 (2)	4 (10)
Cardiac	4 (8)						4 (8)
Stent	3 (9)						3 (9)
Cerebral		1 (2)				1 (2)	2 (4)
Bleeding	1 (1)	1 (1)					2 (2)
Survival	31 (45)	10 (13)	10 (19)	7 (17)	8 (12)	6 (7)	72 (113, 77.4,100^c^)
Retrieved stent	15^b^ (17)	6 (7)	6 (11)	7^b^ (10)	3^b^ (3)	3 (3)	40^b^ (51, 45.1^c^)
Remain stent	19^b^ (28)	4 (6)	4 (8)	3 (7)	6 (9)	3 (4)	39^b^ (62, 54.9^c^)

^a^Tracheoesophageal fistula with esophageal atresia

^b^double-counted patients

^c^% of stent in survival patients

Dramatic respiratory improvement was noted immediately after 144 stent placements in 85 patients. They were rapidly weaned off from respiratory support and discharged home in stable condition. Unfortunately, another two patients, each with one implanted stent, died before hospital discharge due to their underlying severe cardiac failure.

Overall, there were 15 patients expired, mortality rate of 17.2%, with 33 (22.6%) stents still in site. The leading causes of mortality were respiratory failure in 4 patients with 10 stents, and severe cardiac problems in 4 patients with 8 stents. There were 3 stent-related mortalities (3.4%) with 9 implanted stents (6.2%). The first patient was a victim of severe tracheomalacia due to double aortic arch and had a tracheal stent implanted for two years. During the stent retrieval with RE and forceps, severe tracheal perforation occurred and he expired one month later. The second infant who had 3 implanted stents for idiopathic long-segment tracheomalacia. His death might be related to severe stent-associated granulation obstruction, which could not be properly managed in outside hospital due to lack of expertise, and succumbed before he was able to be transferred back to our center. The third was an infant with 5 stents who finally expired in granulation-associated respiratory failure. All these three infants with 9 death-related stents were of *Palmaz* type. Two other patients expired because of their underlying severe cerebral defect and parents refused aggressive management. Lastly, two mortalities were related to massive pulmonary bleeding, one patient having complex cardiac disease with severe pulmonary hypertension, and another with unknown cause of sudden pulmonary bleeding at home who expired in a local hospital. None of these mortalities were directly related to the stent implantation or TFE procedures. All survived patients are free of respiratory symptoms at home except that two children necessitated TFE management once every two months.

### Stent management with TFE

**Fig 5:**
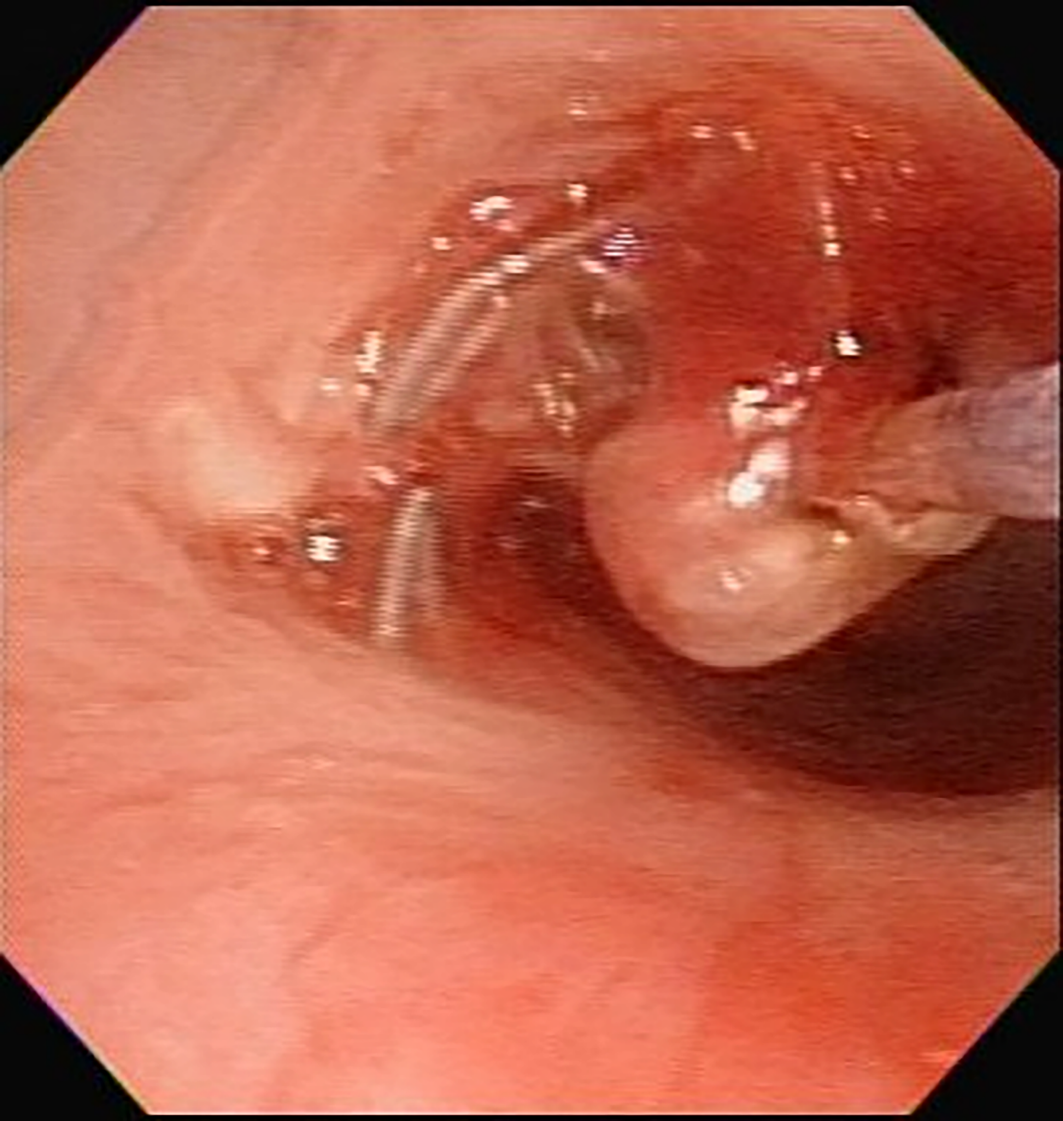
Laser ablation of stent-associated granulation with flexible endoscopy.

**Fig 6:**
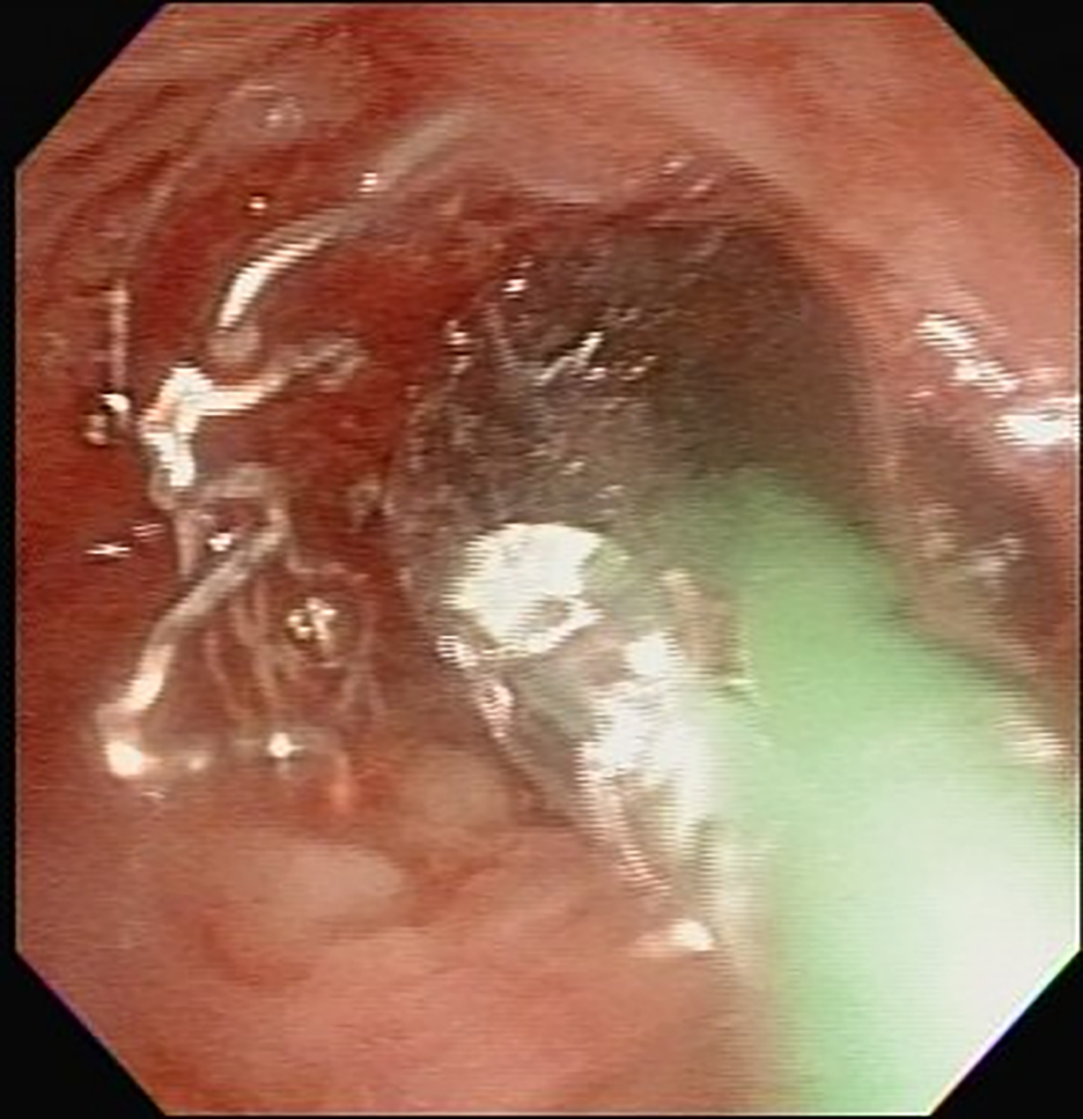
Balloon dilatation plasty. Which could simultaneously compressing stent-associated granulation and expanding the stent with flexible endoscopy.

Major stent-associated problems included obstructive granulation formation, lumen narrowing at the stent ends, infection, stent distortion and fracture. In addition to the medical management, mechanical interventions were performed by TFE with forceps debridement, laser ablation and BDP [12,15–21]. Forceps debridement and laser (Diode, Dornier MedTech®) ablation could remove granulation tissues (Fig 5). BDP was used to decompress granuloma, control local hemostasis, restore and further expand stent lumen to keep patency and remodeling for growing TB lumens. Appropriate angioplasty balloon catheters (Boston Scientific, Natick, MA, USA) with various dimensions were selected, inserted and inflated under direct FE vision (Fig 6). The time of balloon inflation was kept less than 10 seconds to shorten the iatrogenic airway block on ventilation. Repeated BDP might be required to achieve sufficient sizes of lumens. These three TFE techniques could work alone or in combination.

### Stent outcomes and retrieval

**Fig 7:**
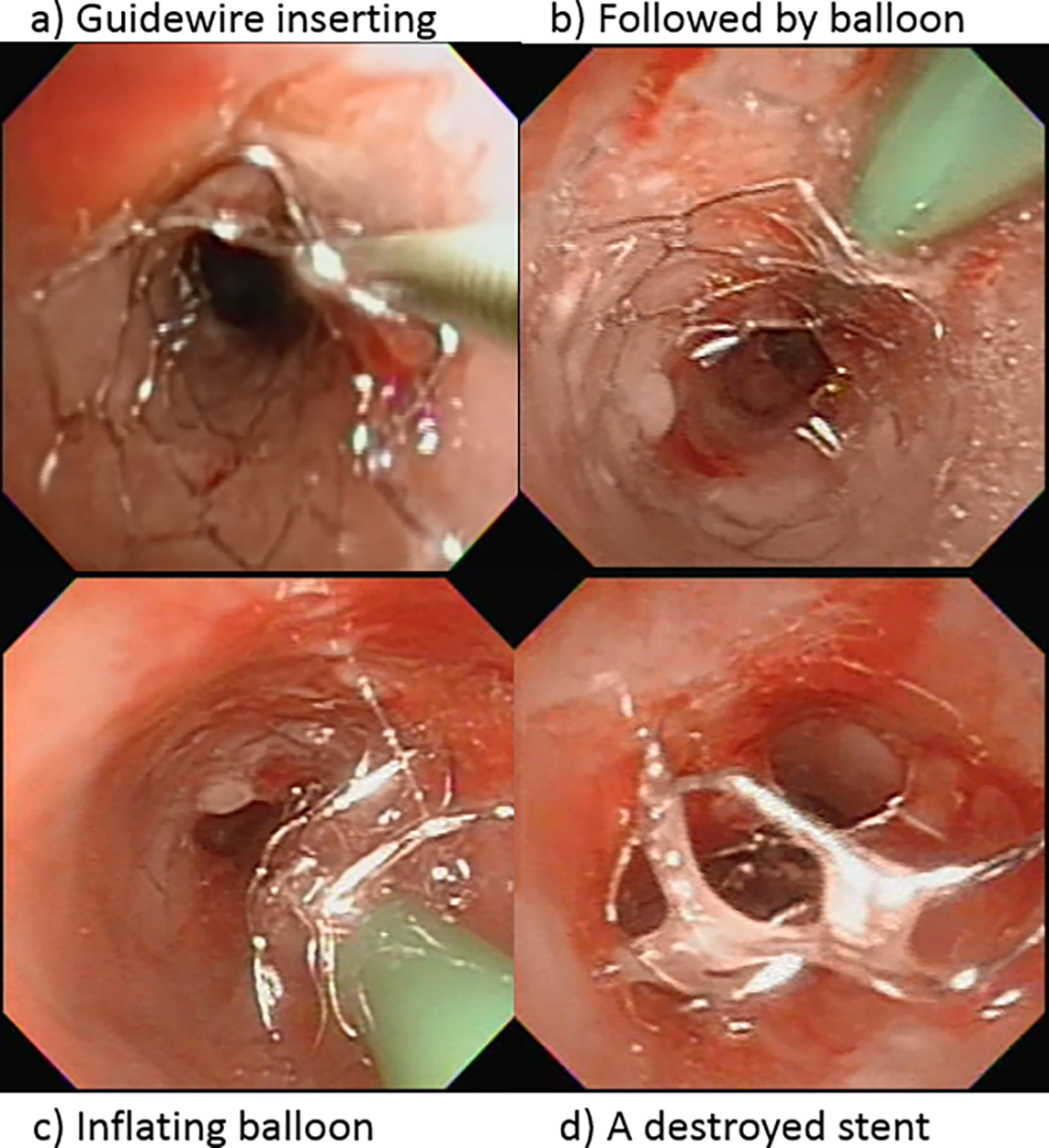
Sequential steps of stent retrieval. (a) Inserting a guidewire in the gap between the stent and mucosal wall; (b) mounting and advancing balloon catheter through the guidewire; c) inflating the balloon to separate the stent from the mucosa; d) the destroyed stent becoming easier for grasping by forceps during retrieval procedure.

At the end of the study, 72 (82.8%) survived patients had 113 (77.4%) stents placed. Sixty-two (54.9%) stents still remained in 39 (54.2%) patients and were maintained functioning with TFE management. The BDP could also be used for retrieving implanted stents (Fig 7). Reasons for not being removed could be divided into two groups. The first group, “Normal group”, included 59 stents without complications, in which 23 stents were fully covered by mucosa and endoscopically invisible but with good patency, 24 stents were visible but unnecessary for removal yet, and 12 stents were visible but parents hesitated for retrieval. The second group, “Difficult retrieval group”, included 2 patients with 3 stents. One patient had 2 stents fully covered by granulation tissues and becoming invisible, which required TFE interventions every two months to keep lumen patency. Another patient had lost medical follow-up for 9 years after a right low bronchial stent placement, but returned with the condition of a total lumen-blocked stent-granuloma. This obstructive lesion could not be approached via RE due to the severe cervicothoracic scoliosis and cardiopulmonary insufficiency.

Both tracheal and bronchial stents could be retrieved (Fig 4B) (Table 4). However, no carina stent had been retrieved yet in this study period, simply because they were all implanted in recent years and still functioning well. In the 72 survived patients, 94 stents were implanted more than two year and might consider removal when indicated. Among them, 51 (54.2%) stents in 40 (55.6%) patients had been retrieved which included 38 whole stents and 13 partial (more than 50%) stents. Reasons for their retrieval included malposition in 4 stents, detached and unneeded in 28 stents, and severely destructed and unrepairable in 19 stents. Retrieving implanted stents usually required greater power of grasping and extracting with forceps. Thus 47 stents were removed with RE and only 4 loose stents with FE. All the retrievals were performed smoothly without significant complications, except one tracheal perforation as mentioned above. Residual stents, which were partial retrieved but destructed, were all appropriately repaired again with BDP.

**Table 4:** Tracheobronchial stent locations, numbers and outcomes (%, *%*)^a^.

Locations	Stent number	Survived patients	Stent number		Survived patients
	Subtotal	Expired^b^	Implanted		Retrieved
			<2 year^c^	≧2 year^d^	
Trachea	84 (57.5, 100)	25 (29.8, *75*.*8*)	5	54 (64.3, *57*.*4*)	35 (41.7, *70*.*2*)
Upper	15	5	2	8	5
Middle	34	12	2	20	17
Lower	35	8	1	26	13
Carina	15 (10.3, *100*)		7	8 (53.3, *8*.*5*)	
Right	7		4	3	
Left	8		3	5	
Bronchi	47 (32.2, 100)	8 (17.0, *24*.*2*)	7	32 (68.1, *34*.*0*)	16 (34.0, *29*.*8*)
Right	18	4	4	10	6
Left	29	4	3	22	10
Total	146 (100, *100*)	33 (22.6, *100*)	19	94 (64.4, *100*)	51 (45.1^e^, *54*.*2*^f^)

^a^(row, column) exclude the column of subtotal

^b^not be retrieved

^c^no attempt to remove

^d^might retrieve if indicated

^e^of 113 implanted and not expired stents

^f^of 94 stents

### Stented durations and final diameters

There are three different stent outcome groups (Table 5). In the expired-patient group of 33 stents, the mean duration was 7.4 ± 9.5 months, which was significantly shorter than the other two survived-patient groups of the retrieved and the remained stents (*p* < .001). Among the expired group, the shortest duration was 10 days. In the retrieved group of 51 stents, the mean duration was 34.9 ± 36.3 months and the shortest duration was 3 days due to malposition. The longest duration of retrieved stent was 184 months, located in the left main bronchus, in a boy with his another tracheal stent already removed 5 years after placement. In the remained-stent group of 62 stents, the mean duration was 82.3 ± 62.5 months.

**Table 5:** Tracheobronchial stent duration (month) and diameter (mm) in different outcomes.

Stent outcomes	Patient Number (%)	Stent		Expanded diameter (mm)		
		Number (%)	Duration (SE) (ranges)	Mean (SE)	Range	*p*
At placement	87 (100)	146 (100)				
Tracheal		84+15^a^		7.2 (1.7)	4–10	
Carinal		15				
Bronchial		47+15^a^		6.5 (1.2)	4–8	
Expired	15 (18.5)	33 (22.6)	7.4 (9.5)			
			(0.3^b^- 35)			
Tracheal		25		8.3 (2.2)	6–15	*0*.*032*
Bronchial		8		6.7 (1.4)	4–10	*0*.*047*
Retrieved	40 (46.0)^c^	51 (34.9)	34.9 (36.3)			*<* .*001*
			(0.1^d^- 184)			
Tracheal		35		9.6 (2.7)	6–15	*0*.*035*
Bronchial		16		7.6 (2.2)	4–9	*0*.*029*
Remained	39 (44.8)^c^	62 (42.5)	82.3 (62.5)			
			(0.9–111)			*<* .*001*
Tracheal		24+15^a^		10.8 (2.3)	5–15	*0*.*018*
Carinal		15				
Bronchial		23+15^a^		9.5 (1.8)	4–12	*<* .*001*

^a^Segment of carinal stent

^b^patient expired 10 days after stent placement due to cardiopulmonary failure

^c^double-counted

^d^retrieved with flexible endoscopy 3 days after placement due to malposition.

At the time of placement, the lumen diameters of the 99 tracheal and the 62 bronchial stents (both included the 14 segments of carinal stent) were 7.2 ± 1.7 (range 4–10, median 8) mm and 6.5 ± 1.2 (range 4–8, median 6) mm, respectively. During surveillance management, stent lumens could be gradually expanded and measured with the BDP technique. Therefore, in the expired group, the lumen diameters of tracheal and bronchial stents were 8.3 ± 2.2 (range 6–15, median 9) mm and 6.7 ± 1.4 (range 4–10, median 7) mm, respectively. In the retrieved group, the diameters of tracheal and bronchial stents were 9.6 ± 2.7 (range 6–15, median 9.0) mm and 7.6 ± 2.2 (range 4–9, median 7) mm, respectively. The latest diameters of the remained 39 tracheal and 38 bronchial stents were 10.8 ± 2.3 (range 5–15, median 10) mm and 9.5 ± 1.8 (range 4–12, median 8) mm, respectively. These stent diameters were significantly increased (p < .001) as correlated with their implanted duration.

## Discussion

This is the first and also the largest report specifically discussing the unique TFE techniques of BEMS implantation, surveillance management and long-term outcomes in pediatric patients. We certainly avoid TB BEMS placement unless absolutely necessary and only when other medical or even TB surgical interventions have failed. There were 11 stents placed in 8 patients as their previous tracheostomy or slide tracheoplasty gave unsatisfactory outcomes. Our results are similar to others using stents for benign TB narrowing that describe dramatic and symptomatic improvement after the restoration of airway lumen but also report more successful retrievals [6–10].

### Benefits of BEMS

There are many commercial types of TB stent with different properties and advantages. Metallic uncovered stents, as the BEMS, are used predominantly for extra-luminal compression. The mesh structure helps to preserve mucociliary function and allows passage of airflow via the struts when placed over branches. There were 22 stents, 15 carinal and 7 bronchial, requiring BDP to further enlarge the interstices over the bronchial orifice. BEMS lumen diameters could also be further expanded appropriately with BDP during surveillance to match the growing lumens without replacement new ones, or simultaneously when BDP were done to compress the associated granulomas or to restore stent structure. In this study, there was no any luminal obstruction happening in the carina or the bifurcated regions.

The initial 20 stents were of *Palmaz* type starting from 1997, but enormous negative experiences were encountered including massive granulation formation and difficult stent retrieval [22–27]. This can be illustrated by our three stent-related mortalities which all happened during this early period. Thereafter, we changed to *IntraStent* for its softer, more elastic and conformable in TB lumen, MRI-compatible [28], as well as being easier for retrieval in piecemeal.

### Advantage of FE with this NIV

In this study, majority of children had already received prolonged endotracheal intubation or PPV prior to stent implantation. These respiratory supports were deliberately shifted to this NIV support. As described above, this NIV can provide both high concentrated inspiratory oxygen and PPV, but without the limitations encountered with face mask, laryngeal mask airway, endotracheal tube and ventilator. In addition, the pressure of PPV can be optionally controlled and adjusted by the bronchoscopist himself. That means, while doing FE with this NIV support, this may preserve original intra-airway morphology and dynamics for accurate diagnosis but also widen TB lumens to facilitate both flexible endoscope and instrument(s) for delicate TFE processes. We have already applied this combination modality in various FE procedures for more than 20 years [12–19].

### Stent placement and surveillance management

Stent placement itself and subsequent management may be difficult because of existing narrowing TB lumen, underlying compromised cardiopulmonary reserve, and lack of appropriate instruments and techniques. Traditional procedures need transportation of patient with respiratory support equipment, RE, fluorography, general anesthesia or even more invasive surgeries that may further jeopardize the risky status and make these interventions more difficult or impossible. Though there have been mentioned in literatures about the notable problems of the metallic stents such as granulation formation, issues of repairability, expandability and retrievability [22–27], we have gradually adopted a more proactive strategy over the last two decades of BEMS usage with regular FE surveillance and effective management before the problems have worsened significantly. Medical managements are given with daily saline nebulizer, steroids inhalation, mucolytics and anti-gastroesophageal reflux based on individuals’ conditions. FE surveillance includes regular FE check of whole airway once every two to six months with immediate actions via appropriate TFE, if indicated.

In our TFE techniques, short working-length FE is easily handled, has enough length for managing TB stents in children, and provides direct and dynamic visualization of full approachable airways, which are better than other techniques of classical long-length FE, fluorography or RE. Patients can be appropriately sedated and supported with this NIV skill without requiring any artificial bag, mask, RE or endotracheal tube. In addition to handling the FE and associated instrument(s), the bronchoscopist could also easily, simultaneously and optionally provide and control the intensity of NIV-created PPV for dynamic expansion of target airway lumens. This may be an excellent modality for detailed assessment of airway, placing BEMS and managing stent related problems. We have found that these BEMS could be accurately placed and well managed to maintain TB lumen patency with TFE.

### Stent retrieval

Those stents, which are nonfunctioning, destructed beyond repair, or associated with serious complications, should promptly be retrieved. In addition, we recommend that those stents placed more than two years may be the potential candidates for retrieval. Over-epithelialization which may lead to stents becoming invisible, they may be presumed to be permanent in TB wall when work functionally. Otherwise, these implanted and invisible stents may difficulty in subsequent removal [22–27, 29] and therefore may need open surgery. In our series, only 2 stents in one patient has become this condition [22]. There were 51 stents, 35 tracheal and 16 bronchial stents, all of which could actually be retrieved by using rigid endoscopy when they were well visible with FE and no longer needed. However, before the retrievals with RE, extra preparatory works were needed to be done in some cases to separate and destroy the target stents from the underlying mucosa by TFE, as described in Fig 7, for the subsequent easier extraction in either fragments or whole pieces. Those un-retrieved or residual portions were left there after appropriate repair with TFE of balloon stent plasty.

### Clinical implications

Traditionally, because of significant disadvantages of using metallic stent, a black box warning from the United States Food and Drug Administration has been issued which recommended that metallic stents should preferably not be used for benign diseases unless absolutely necessary [30]. From this retrospective study, we recognize that the BEMS do have benefit when used in TB narrowing on carefully selected pediatric patients. It is also encouraging that the vast majority of our survival patients have achieved asymptomatic lives from respiratory perspective. The TFE with NIV support for BEMS placement, regular surveillance, adjuvant management, appropriate dilatation and finally retrievals have provided a safe and valuable strategy which have altered and expanded the clinical utilization of metallic stents in pediatric field.

Children who have TB stents placed certainly require appropriate follow-up which preferably may be a combination of clinical, radiological and endoscopic assessments in a special tertiary care center. The bronchoscopists who implant the stent should also be capable of maintaining and finally retrieving the stents. Therefore, they should be well trained, experienced and skillful in various TB stent procedures of both TFE, RE and NIV performance. Advances in new stent design of more durable, easily retrievable or absorbable ones [31,32] may augment its clinical applications. Further multi-center prospective studies are the best approach to evaluate their definite effectiveness and efficacy.

### Limitations

There are some limitations in this study. It is a retrospective study from a single institution with no control group for comparison. Detailed description on the techniques of TFE and NIV of TB BEMS placement and subsequent management such as laser ablation, BDP and stent retrieval are beyond the theme of this study, but they were discussed in our previous published papers [15–21].

## Conclusions

BEMS may provide safe, effective and less-invasive therapy for internal support of severe TB narrowing in selected pediatric patients. Short working-length FE with this NIV is a versatile technique for placement and surveillance management of forceps debridement, laser ablation and BDP to immediately maintain and improve stent function and therefore quality of life. Finally, these implanted BEMS could be successfully retrieved by FE or RE once they are no longer indicated. With more skills and competency of these procedures, the BEMS may become more valuable in pediatric patients.

## Supporting information

S1 Video Placing a tracheal stent. Add a tracheal metallic stent in a severe tracheomalacia infant who already has a right carina stent inside for correct his peri-carina and right main bronchial malacia.
(MP4)
